# Delivery of Various Cargos into Cancer Cells and Tissues via Cell-Penetrating Peptides: A Review of the Last Decade

**DOI:** 10.3390/pharmaceutics13091391

**Published:** 2021-09-02

**Authors:** Alireza Shoari, Raheleh Tooyserkani, Mehdi Tahmasebi, Dennis W. P. M. Löwik

**Affiliations:** 1 Department of Medical Biotechnology, Faculty of Medical Sciences, Tarbiat Modares University, Tehran 14115-111, Iran; alireza.shoari@modares.ac.ir (A.S.); ra.tooyserkani@modares.ac.ir (R.T.); mh_tahmasbi@modares.ac.ir (M.T.); 2 Bio-Organic Chemistry, Institute for Molecules and Materials, Radboud University Nijmegen, Heyendaalseweg 135, 6525 AJ Nijmegen, The Netherlands

**Keywords:** cell-penetrating peptides, drug delivery, cancer, cargos, therapeutic molecules

## Abstract

Cell-penetrating peptides (CPPs), also known as protein transduction domains, are a class of diverse amino acid sequences with the ability to cross cellular membranes. CPPs can deliver several bioactive cargos, including proteins, peptides, nucleic acids and chemotherapeutics, into cells. Ever since their discovery, synthetic and natural CPPs have been utilized in therapeutics delivery, gene editing and cell imaging in fundamental research and clinical experiments. Over the years, CPPs have gained significant attention due to their low cytotoxicity and high transduction efficacy. In the last decade, multiple investigations demonstrated the potential of CPPs as carriers for the delivery of therapeutics to treat various types of cancer. Besides their remarkable efficacy owing to fast and efficient delivery, a crucial benefit of CPP-based cancer treatments is delivering anticancer agents selectively, rather than mediating toxicities toward normal tissues. To obtain a higher therapeutic index and to improve cell and tissue selectivity, CPP-cargo constructions can also be complexed with other agents such as nanocarriers and liposomes to obtain encouraging outcomes. This review summarizes various types of CPPs conjugated to anticancer cargos. Furthermore, we present a brief history of CPP utilization as delivery systems for anticancer agents in the last decade and evaluate several reports on the applications of CPPs in basic research and preclinical studies.

## 1. Introduction

**Figure 1:**
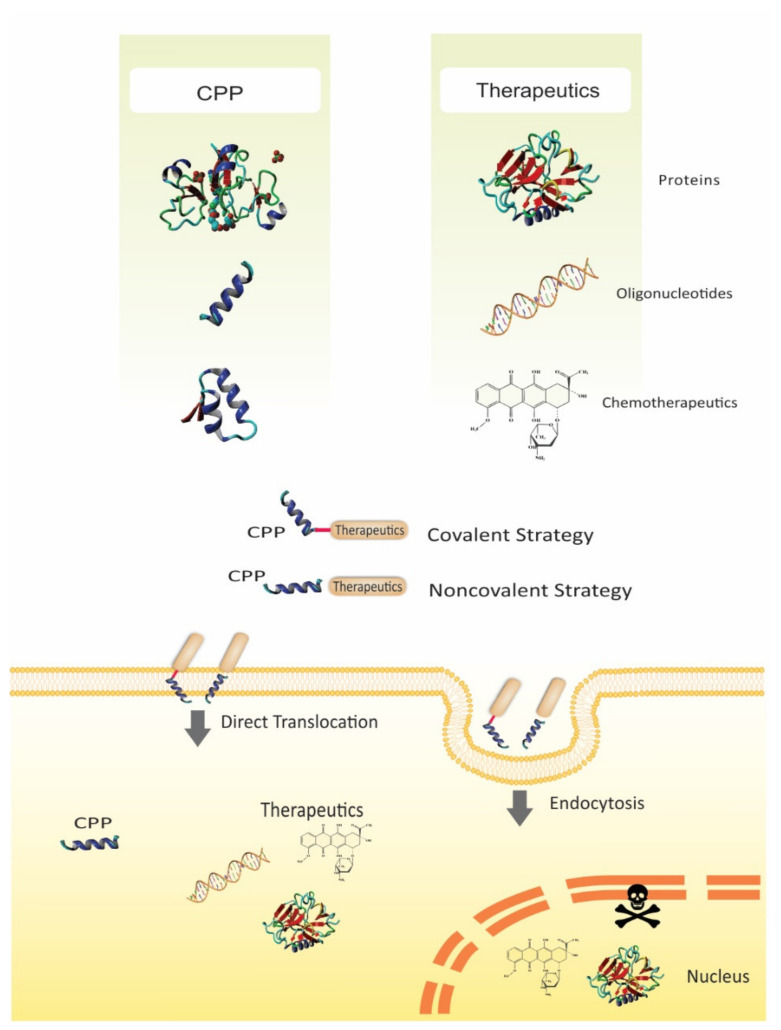
Schematic representation of recent CPP-based strategies. The hydrophilic nature of cargos such as nucleic acids, small drugs, proteins, or peptides, can hinder their cellular uptake and subsequent intracellular localization. Conjugating the cargos to a CPP via noncovalent interactions or covalent bonds facilitates the CPP-conjugated therapeutic to cross the cell membrane and reach intracellular areas which are otherwise challenging to access, thus improving the therapeutic efficacy.

Globally, with one in every six deaths, cancer is the second leading cause of death, and in 2018, approximately 9.6 million deaths were reported to be cancer-related [1]. In the past decades, multiple endeavors have been undertaken to discover novel therapies to combat cancer; however, several hurdles, such as non-selective cell targeting, the emergence of drug-resistance and inefficient drug delivery, should still be overcome [2]. The impermeability of cellular membranes does not allow large protein complexes, genetic material and many other small molecules to enter, which explains the inability of drugs to find their ways across the plasma membrane into the cytosol [3]. Therefore, numerous studies have investigated the impact of implementing drug delivery systems, including viral-based vectors, nanoparticles and cell-penetrating peptides (CPPs), to improve cell penetration [4]. Some of the first studies that led directly to the discovery of CPPs were published in 1988 [5]. CPPs, formerly known as protein transduction domains (PTDs), are a class of 5 to 30 residue peptides with the capability to pass through biological membranes in an energy-dependent or independent manner [6]. Frankel and Pabo discovered that the TAT protein (Trans-Activator of Transcription) in human immunodeficiency virus-1 (HIV-1) possesses the ability to cross plasma membranes [5]. Since this first report, over 1700 CPPs have been categorized and registered in the CPPsite 2.0 database [7]. Lately, in an interesting study, screening the proteome of the severe acute respiratory syndrome coronavirus 2 (SARS-CoV-2) disclosed a total of 310 CPPs. Whereas 64% of these CPPs had immuno-modulatory attributes, 22% were identified with anti-cancer properties [8]. Experimentally, CPPs have demonstrated to be able to deliver small and large bioactive cargos into cells both in vitro and in vivo [9]. The variety of targeted cell types reveals the seemingly limitless applicability of CPP-based therapies for the treatment of numerous diseases including heart diseases, pain, inflammation and cancer [10]. However, no CPP-conjugated drug has yet been approved by the US Food and Drug Administration (FDA), and only a few have been evaluated in clinical trials [11]. This might be attributable to some of the unfavorable characteristics of CPPs including low cytosolic delivery efficiency, susceptibility to proteolytic degradation, and the lack of selectivity for tumor cells and tissues [12,13]. To efficiently deliver anticancer drugs to desired cells, several methods have been utilized based on active targeting strategies [14,15]. To achieve selective delivery to cancer cells, diverse approaches have been implemented [16], for example, antibody−drug conjugates (ADCs) [17,18] or peptide−drug conjugates (PDCs) [19] where the ADC or PDC displayed selective uptake via the cancer cells. In most cases, there is receptor-mediated endocytosis for selective uptake [20]. Five ADCs [21] and a two PDCs [22,23] are now FDA-approved for cancer therapy. With the mentioned capacity of CPPs to pass different cargos into cells with restricted toxicity, they are now recognized as promising tools for both basic research and clinical studies [24]. CPPs can deliver a variety of cargos including nucleic acids, therapeutic proteins/peptides and chemotherapeutic agents [25]. CPPs can be classified into cationic and amphipathic types based on their chemical and physical attributes [26]. Short amino acid sequences that generally contain histidine, lysine and especially arginine are characteristics of cationic CPPs. Such amino acids harbor cationic charge, which is required to establish interactions with the plasma membrane’s anionic motifs in a receptor-independent fashion [27]. On the other hand, amphipathic CPPs contain both hydrophilic and lipophilic amino acids to mediate the peptide translocation across the plasma membrane [28]. Additionally, CPPs are also categorized based on their source; (1) natural protein-derived CPPs, such as Penetratin (Pen) and Tat, (2) chimeric CPPs such as transportan, comprising 14 amino acids from mastoparan (*Vespula lewisii* wasp venom) and 12 from the N-terminal part of the neuropeptide galanin and (3) completely synthetic CPPs such as oligoarginines and peptide nucleic acids (PNAs) [29,30]. The conjugation of CPPs to their cargos can be established via covalent (cleavable or non-cleavable) as well as non-covalent interactions. Both methods have their advantages and disadvantages, though selection of a particular method might depend on the specific structures of both the CPP and the cargo. Conjugation can be easily achieved through direct mixing of the CPP and cargo [31]. Since the emergence of CPPs more than 25 years ago, the number of scientific publications demonstrating the use of CPPs for delivery of various cargos has been growing [32]. Different investigations have demonstrated the potential of CPPs as promising tools for co-delivery of drugs and genes to combat drug resistant tumors [33]. The combination of CPPs with nanoparticles has been investigated in order to fill the gap between both molecules and to potentiate the progress of a novel compound/conjugate that holds improved effectiveness, accuracy and therapeutic function. The decorated nanocarriers holding the anticancer cargos with the CPPs can enable targeted treatment and obliteration of tumors without influencing normal tissues. Numerous investigations demonstrated that the conjugation between CPPs and nanoparticles is a potential delivery system in cancer cell-lines and cancer animal models [34]. Recently, results of an increasing number of clinical trials using CPPs have been properly discussed by Vale et al. [35]. During the last decade, the role of CPPs in cancer therapy has been evaluated and reviewed in several studies [36,37]. This review covers the main aspects of CPP-based cargo delivery with a focus on cancer therapy and describes the very latest promising CPP-based anticancer therapy strategies. Here, we try to provide a comprehensive classification of various cargos delivered by CPPs as well as their biomedical applications. Particularly, we summarize various CPP studies focused on the delivery of cargos into tumor cells as a treatment strategy (Figure 1). Hence, we hope this review will contribute to the understanding of CPP-based anti-cancer drug delivery and guide the readership through the latest achievements made in this field.

## 2. Challenges of Biomacromolecules and Chemotherapeutics Delivery

After decades of concentrated studies, therapy with genetic materials has become one of the most encouraging approaches for treating cancer. Nevertheless, the lack of universal delivery systems has hindered the clinical utilization of gene therapy, regardless of its tremendous potential [38]. Furthermore, gene therapies are required to be tissue-specific, and nucleic acid drugs need to penetrate the intracellular lumen to play a role in the nuclear machinery with no substantial toxicity [39]. Delivering nucleic acids into cells is a problematic mission since they are susceptible to enzymatic degradation as well as naturally having high molecular weight and being anionic, which makes them weak translocators of the cell membrane [40]. One of the strategies to deliver genetic materials to the host cells is utilization of viral vectors [41]. However, non-viral gene delivery systems attend as a substitute to viral gene vectors due to their advantages such as nearly no immune response and relatively low toxicity [42]. Other potential advantages of these systems comprise cell-type specificity after being linked with a targeting ligand, simplicity in preparation and capability of dealing with large plasmid DNA. There are four barriers that must be overcome by non-viral vectors to attain effective gene delivery. The vector must be able to firmly condense and defend DNA, target specific cell-surface receptors, disrupt the endosomal membrane and deliver the DNA cargo to the nucleus [43]. Peptide-based vectors such as CPPs are valuable over other non-viral approaches in that they are capable to attain all four of these aims [27].

Molecular drugs based on proteins or peptides gained specific attention due to their potency and specific mode of action which resulted in fewer side-effects and predictable responses when compared to conventional small molecule drugs [44]. The hydrophilic nature of proteins and peptides renders them impermeable to cell membranes. Therefore, to effectively deliver protein and peptide-based medications across the endothelial and epithelial barriers or plasma membrane, a permeation-improving approach must is required [12]. As another main disadvantage, biopharmaceuticals are presently mostly administered via injection, which are accompanied with discomfort and pain in patients, therefore often causing poor patient admission [45]. A membrane permeation-improving approach may also be appropriate for drugs dosed by a non-injectable method of administration (e.g., pulmonary, nasally, orally), where cells in the form of a firm epithelium must be crossed to get access to the systemic circulation and consequently a target receptor [46]. Lastly, also protein- or peptide-based drugs that play a role in the brain may profit from a formulation approach which enables them to cross blood−brain barrier (BBB) [47].

Chemotherapy for cancer treatment still suffers a lot of deficiencies due to the toxicity of the chemotherapeutics to healthy normal cells and also to resistance advanced via tumor cells to the anticancer medication [48]. The main problem with cancer chemotherapeutics is the lack of selectivity to tumor cells and because of that a less effective antitumor effect. Hence, to deliver a chemotherapeutic intact to the cytosol of every cancer cell and not to normal ones is the main challenge in cancer therapy [2]. Physio-chemical properties of chemotherapeutics, including size and surface charge, hydrophilicity and poor solubility significantly impact the effectiveness of chemotherapy. Moreover, high liver accumulation, weak bioavailability and rapid renal clearance can render chemotherapy far from unsuccessful [49].

Recent advancements and developments in CPPs have uncovered their powerful ability to overcome the above-mentioned drawbacks in the delivery of biomacromolecules and chemotherapeutics, which we have reviewed in the sections below.

## 3. Delivery of Nucleic Acids

Oligonucleotide (ON)-based medications have faced incremental demands for the treatment of numerous human genetic disorders because of their remarkable capability to specifically modulate gene expression [50]. However, their clinical translation has often been hindered due to poor biodistribution [51]. In this regard, CPPs have emerged as an opportunity to enhance the cellular delivery of nucleic acids as non-permeant biomolecules (Table 1) [52]. Novel strategies have been developed based on amphiphilic modulation of cationic peptides via, e.g., a hydrazone bond to deliver nucleic acids efficiently [53]. Moreover, the simplicity and specificity of the CRISPR/Cas9 technique allows researchers to directly target and edit certain loci in the cell genome without the usage of protein engineering [54].

**Figure 2:**
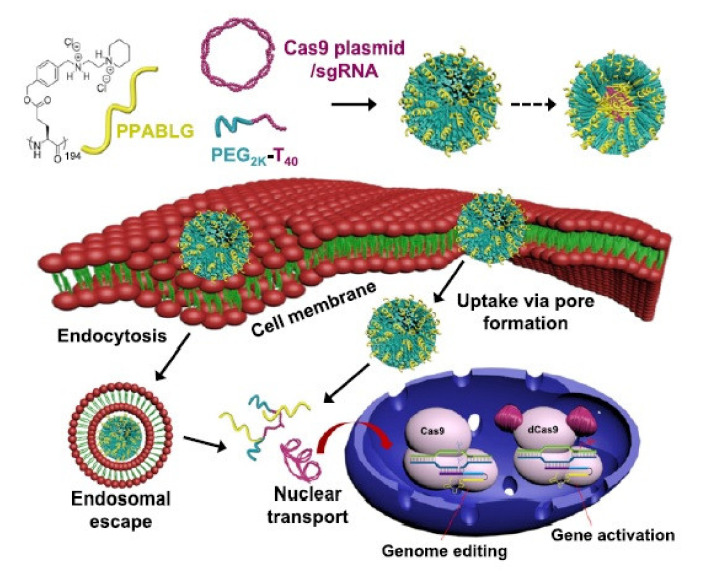
Schematic illustration showing the formation of P-HNPs and the intracellular activity of Cas9 expression plasmid/sgRNA in performing genome editing or gene activation. Adapted with permission from [56], United States National Academy of Sciences, 2018.

Recently, Seijo et al. described an amphiphilic penetrating peptide, named PT24, as the first supramolecular strategy for the direct delivery of Cas9 using a penetrating peptide vehicle. This peptide was generated through hydrazone bond formation between a hydrophobic aldehyde tail and the cationic peptide scaffold. Furthermore, they conducted a single incubation step gene-editing process in HeLa and particular human lung cancer cell lines to evaluate the functionality of this peptide as a carrier. The results indicated the amphiphilic carrier peptide can deliver Cas9 with low toxicity and acceptable efficacy [55]. In the same year, Wang et al. used polyethylene glycol (PEGylated) nanoparticles (named P-HNPs) based on the α-helical polypeptide PPABLG (poly(γ-4-((2-(piperidin-1-yl)ethyl)aminomethyl)benzyl-l-glutamate)) for efficient delivery of sgRNA and Cas9 expression plasmid to several cell lines (Figure 2). The cell-penetrating α-helical polypeptide improved pCas9 and/or sgRNA cellular internalization and endosomal escape. With up to 60% Cas9 transfection efficiency and 67% sgRNA uptake efficiency, the use of the P-HNPs was demonstrated to be beneficial in comparison with other currently used polycation-based gene delivery systems. In tumor cell lines such as K562 and HeLa cells, P-HNPs exhibited 11% and 33% improved uptake efficiency, respectively.

**Figure 3:**
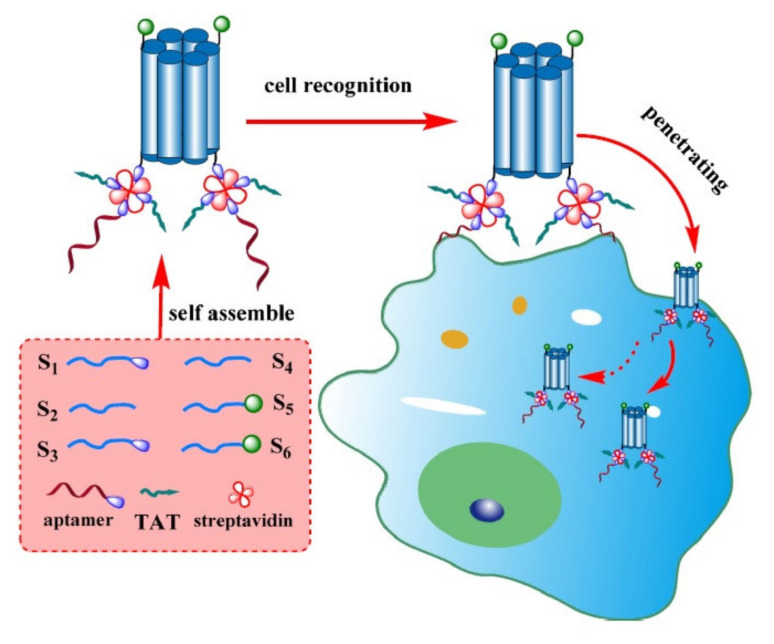
Schematic illustration of DNA nanopore construction and tumor cell recognition. Adapted with permission from [59], Springer, 2017.

With 35% gene deletion of the polo-like kinase 1 (Plk1) gene and a decline in the Plk1 protein level, Wang et al. demonstrated that efficient delivery of Cas9 plasmid/sgRNA to HeLa tumor tissues can be achieved using PHNPs, which can consequently inhibit tumor growth. The efficient delivery of this gene-editing platform via the mentioned system, as indicated by the results from several cell lines and in vivo experiments, represents a qualified method for therapeutic applications and biological research [56]. Due to the presence of particular targets on the surface of tumor cells and the obvious effects of selective cancer treatments, tumor-specific targeting is commonly used as a therapeutic approach against various cancers [57]. Recently, DNA nanostructures have been recognized as novel nanomaterials demonstrating considerable biocompatibility and low cytotoxicity which can vouch for their application in tumor cell detection and drug delivery [58]. Guo et al. designed a DNA nanopore composed of six DNA duplexes functionalized with Ramos cell-specific aptamers (human Burkitt’s lymphoma) and the TAT protein as the CPP (Figure 3). In detail, the nanopore structures attached to the cell surface and subsequently internalized into cells with the help of CPPs. This modified DNA nanopore has several advantages over the unmodified form, including efficient targeting specificity towards Ramos cells and enhanced cellular uptake. This structure also represents a suitable platform for the development of targeted anti-cancer therapies with low cytotoxicity [59].

Increasing the expression of therapeutic genes or cancer-associated gene knockdown as a gene therapy strategy holds a great promise for the treatment of different cancers. In this regard, small interfering RNA (siRNA) appears as a suitable RNA-based gene therapy module that can be directed towards cancer treatment. However, efficient delivery vectors are needed for the transfer of genetic cargos to their intracellular action sites within tumor cells [60].

With the combination of the TAT peptide, the MCF-7-targeting peptide DMPGTVLP, and cationic liposomes, Wan et al. designed an effective gene-delivery system for targeting breast cancer cells with enhanced cell-specific internalization and effective escape from endosomes. This new formulation demonstrated enhanced gene silencing and expression compared with the peptide alone. Moreover, in contrast to the commercial siRNA delivery agent, Lipofectamine^®^ RNAiMAX, this peptide/lipid hybrid system exhibited higher gene knockdown efficacy. Furthermore, in a wound-healing assay, the delivery of B-cell lymphoma 2 (Bcl-2) siRNA to MCF-7 cells suppressed cell migration via the complete knockdown of the Bcl-2 gene [61]. In 2016, Golan et al. designed an efficient siRNA intracellular delivery system through the combination of octaarginine (R8), a subunit of the influenza virus hemagglutinin (HA2), and fluorescein-5-isothiocyanate (FITC)-labeled HPMA (N-2-hydroxypropyl methacrylamide) copolymer which also exhibited high serum stability and low in vitro cytotoxicity. The complex demonstrated improved intracellular siRNA delivery and endosomolytic activity as a result of the multivalent nature of R8 in the polymer. Furthermore, by disrupting the endosomal membranes, the endosomal escape of this compound led to a considerable reduction in the mRNA levels of oncogenic *RAC1* (Rho small GTPase proteins) in human ovarian adenocarcinoma and lung carcinoma cells [62]. DNA, as a cargo, always faces numerous disadvantages such as insertional mutagenesis and the need for nuclear entry, while there is a poor probability for an mRNA to be incorporated into the host genome [63]. Nevertheless, mRNA inherently has a high degree of susceptibility to nucleases besides having an inadequate level of protection in biological environment [64]. To overcome this drawback, Chen et al. developed an inspiring polymeric micelle-based delivery system via the combination of cyclic Arg-Gly-Asp peptides (cRGD), PEG and poly(N-isopropylacrylamide) (PNIPAM) polymer. In particular, the ionic core of the complex neutralizes the negative charge of the mRNA molecules, while the redox-responsive disulfide crosslinking mediates the complexation of the mRNAs into a nano-sized structure which can prevent premature mRNA structural disassembly in harsh biological environments. Furthermore, the cRGD ligand conjugated to the formulation exhibited enhanced tumor accumulation, improved cellular uptake in U87 cells (overexpressing α_V_β_3_ and α_V_β_5_ integrins) and enhanced gene expression that contributes to better mRNA survival in a biological environment [65].

Lactaptin, a human milk kappa-casein protein with a molecular weight of 8.6 kDa, and a recombinant analog of lactaptin, RL2, were recently introduced as molecules with cytotoxic activity against mammalian cancer cells [66]. It has been demonstrated that RL2 is capable of entering both human cancer and non-malignant cells and attaching to cytoskeletal structures. In a more recent study, Chinak et al. evaluated the potency of RL2 as a gene delivery system for nucleic acids, composed of a complex of RL2 and green fluorescent protein (EGFP)-expressing plasmids. Delivery potency of this formulation in epidermoid carcinoma and lung adenocarcinoma cells was evaluated by fluorescence microscopy. The expression of EGFP in treated cells strongly confirmed the presence of the penetrating plasmid DNA. Alternatively, RL2:siRNA complexes significantly suppressed the EGFP expression as the siRNA against EGFP was efficiently delivered into lung adenocarcinoma cells [67].

In 2011, El Andaloussi and colleagues designed PepFect 6 (PF6) by covalently conjugating a novel chloroquine analog to transportan10 (TP10). This formulation helped PF6 to escape acidic endosomal compartments and subsequent lysosomal degradation by delaying the acidification of the endosomes and osmotic swelling. Interestingly, siRNA-mediated gene knockdown in various tissues was successfully achieved by utilizing the HPRT1 housekeeping gene as a target following the systemic administration of PF6/HPRT1-siRNA into mouse animal models. In the same year, this group also introduced PepFect 14 (PF14), which is an amended version of a previously described peptide, stearyl-transportan10 (stearyl-TP10), in which leucines and ornithines were used rather than isoleucines and lysines, for the efficient delivery of 2’-O-Me oligoribonucleotides, as splice-correcting oligonucleotides (SCOs), to various cell lines. PF14 exhibited significant SCO-mediated splice-correction in serum-containing and serum-free media in mdx mouse myotubes and HeLa pLuc705, even greater than that of the commercially-available lipid-based vector Lipofectamine^TM^ 2000 (LF2000). In addition, the efficiency and stability of this formulation were enhanced using a solid dispersion technique with promising results [68]. Numerous CPPs have also been employed to deliver ONs via non-covalent complexation approaches because of their high versatility and simplicity [69]. Nevertheless, to improve membrane interactions upon non-covalent complexation and to increase the capability of nanoparticle formations, further modifications on CPPs are required [70]. In 2012, the same group at Stockholm University demonstrated that PF14, when complexed with hypoxanthine phosphoribosyl transferase (HPRT1) and firefly luciferase targeting siRNAs, exhibited efficient delivery and effective splice correction activity in several human tumor cell lines. Moreover, the nanocomplexes exhibited sufficient activity and stability of RNAi-mediating responses after incubation in highly acidic environments of simulated gastric fluid [71].

**Figure 4:**
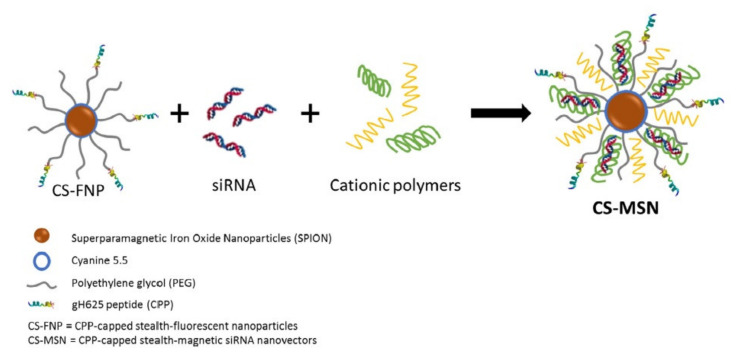
Scheme of CS-MSN and its components, CS-FNP, siRNA and cationic polymers. Adapted with permission from [76], Elsevier, 2018.

To accomplish gene-based therapy for the treatment of brain tumors, ONs need to traverse the blood−brain barrier (BBB) [72]. For a brain-targeted drug delivery system, one of the promising options is homing peptides combined with receptor-mediated transcytosis [73]. Derived from the Kunitz domains of aprotinin, Angiopep-2 (ANG) is a 19-aa oligopeptide that can bind to low-density lipoprotein receptor-related protein-1 (LRP1) and penetrate through the BBB [74]. Recently, Srimanee et al. modified PF14 and PF28 by applying both covalent conjugation and non-covalent complex formation to achieve more selective targeting of glioma along with an enhanced gene-silencing efficacy. They developed highly stable and non-toxic complexes of PF14 with hexaglutamate-modified ANG (PF14:TG1) which were used for siRNA delivery to the human glioblastoma cells U87, and exhibited encouraging results as novel carriers [75]. Ben Djemaa and colleagues developed a novel drug delivery system that contained Ac-HGLASTLTRWAHYNALIRAFC-CONH_2_ as the CPP (named gH625), PEG and iron oxide nanoparticles named as CS-FNP (Figure 4). Furthermore, to protect this complex from degradation and to increase internalization into cells, cationic polymers such as chitosan and poly-l-arginine were used to bind anionic siRNAs. This formulation improved the blood circulation duration, bioavailability and internalization of anti-GFP siRNAs into triple-negative breast cancer cells [76].

To develop a gene carrier with high cellular uptake efficacy and intracellular release suitable for systemic administration, Tanaka et al. synthesized a drug delivery system comprising the cytoplasm-responsive peptide CH2R4H2C conjugated to methoxypolyethylene glycol-polycaprolactone polymers. In non-reducing environments such as blood and the extracellular space, this complexed carrier peptide can form strong complexes with ONs as a result of the formation of intermolecular disulfide cross-linkages between cysteine (C) residues as well as ionic interactions. The carrier/anti-vascular endothelial growth factor siRNA (siVEGF) complexes exhibited considerably lower cytotoxicity, greater uptake efficiency and early endosomal escape and, as a result, stronger silencing effects compared to naked siVEGF in S-180 sarcoma cells. They also exhibited remarkable antitumor effects in S-180 tumor-bearing mice [77].

One of the main oncogenes in human ovarian cancer is inhibitor of DNA binding 4 (ID4). In this regard, Ren and colleagues synthesized a complex delivery system containing the cyclic nonapeptide LyP-1 (CGNKRTRGC)-siRNA targeting GFP (siGFP) to evaluate the applicability of ID4 as a therapeutic target of ovarian cancer. In particular, LyP-1 was selected from a library of tandem peptides capable of tumor-homing and penetration. Owing to the considerable ability of LyP-1 to bind the overexpressed mitochondrial/cell surface p32 protein, the formulation exhibited high translocation and tissue-penetrating properties as well as enhanced gene silencing capability in HeLa cells expressing the destabilized GFP. The treatment of ovarian tumor-bearing mice delivering ID4-specific siRNA inhibited the growth of established tumors and considerably enhanced survival [78]. Five years later, the same group used this peptide-based delivery system to deliver siRNA into primary human vestibular schwannomas (VSs), a primary intracranial tumor of the myelin-forming cells of the vestibulocochlear nerve. In brief, they designed a peptide containing Myristoyl, TP and iRGD. which was complexed with the tumor necrosis factor-alpha (TNF-α)-siRNAs to target primary human VS cells that overexpress αvβ3/β5 integrins. They reported selective internalization of this formulation into the cytoplasm of these cells in addition to substantial inhibition of the TNF-α gene expression [79]. 

To achieve prolonged siRNA circulation and tumor accumulation as well as to promote internalization into tumor cells, protease-responsive nanoparticles have recently gained a considerable deal of attention [80]. The degradable nanoparticles were developed by introducing a tumor acidity-responsive PEGylated anionic polymer on the surface of positively-charged polycation/siRNA complexes by electrostatic interactions [81,82,83]. In a majority of tumors, matrix metalloproteinase 2 (MMP-2) is an overexpressed protease that digests the extracellular matrix [84]. Several studies have revealed that MMP-2-triggered degradable nanoparticles can improve transfection efficiency by enhanced cellular uptake and endosome escape. Using this approach, Wang et al. developed R9-based micellar nanoparticles with MMP-2-responsive peptides to form a “Micelleplex” for the delivery of Plk1-targeting siRNAs. They indicated that the MMP-2-responsive nanoparticles exhibited enhanced SiRNA delivery to MDA-MB-231 cells, potentially inhibiting breast cancer growth upon systemic injection in MDA-MB-231-bearing xenograft mice [85].

Animal viruses destabilize the host cell endosomal membranes by exploiting proteins with endosome-disruptive fusion peptide domain sequences, thus resulting in efficient unloading of the viral genome into the cytoplasm [86]. The acidification step in this process plays a vital role in the destabilizing endosomal membranes and has been mimicked using synthetic peptides named fusogenic peptides [87]. Fusogenic peptides have mediated cytosolic delivery of siRNAs and have also resulted in enhanced siRNA-mediated silencing effects; however, these peptides harbor certain disadvantages. First, separation of peptides from siRNA cargos due to peptide co-localization in different endocytic vesicles is probable. Second, releasing siRNA cargos is possible, while peptides are entrapped within vesicles [88]. To overcome these issues, Cantini and colleagues developed a chimeric peptide containing an INF-7 peptide as the fusogenic sequence linked to a cationic nona(d-arginine) peptide as a highly efficient CPP that can firmly bind siRNAs and enhance intracellular delivery and endosomal escape. This formulation exhibited efficient therapeutic silencing of the *CIP2A* (Cellular Inhibitor of PP2A) oncogene in human tongue squamous cell carcinoma and reduced the invasiveness and anchorage-independent growth of oral cancer cells [88].

Delivery of siRNAs in vivo, particularly to dysfunctional tumor sites and endothelial cells, continues to be a main challenge in developing anti-cancer therapy. In vivo phage panning is a functional method to select tumor-targeting CPPs [89]. Designing such targeted ligands requires utilizing multiple tumor-specific markers, expressed on the surface of tumor cells. Since the growth and metastasis of most primary tumors depend on the overexpression of vascular endothelial growth factor receptor-1 (VEGFR-1) as a crucial protein, VEGFR-1 can be considered a suitable target for the development of tumor-targeted CPPs [90]. One of the novel tumor-targeted peptides with high affinity to VEGFR-1 is a six amino-acid peptide called A1 (WFLLTM) which has been selected through phage library screening methods [91]. Additionally, a new tumor-targeted CPP has been designed by Fang and colleagues via the conjugation of A1 to TAT peptides. The in vitro selectivity of FITC-labeled TAT-A1 complexes were evaluated in human hepatocarcinoma HepG2cells using laser confocal microscope. It was reported that TAT-A1 complexes exhibit more efficient penetration into VEGFR-1-overexpressing HepG2 cells. Moreover, the internalization efficiency of TAT-A1-GAPDH siRNA into human hepatocarcinoma cells was considerably higher in comparison with TAT and 50-FAM-labeled siRNA [92]. In addition, in 2018, Lee et al. developed cancer-specific CPP carriers, called BR2 (RAGLPFQVGRLLRRLLR), to selectively deliver anti-vascular endothelial growth factor siRNAs (siVEGF) to particular cancer cells. The results indicated higher levels of siVEGF internalization into human colon cancer cells and HeLa cells leading to a significant reduction in the intracellular VEGF levels. Furthermore, via BR2-(siGFP), they evaluated target gene silencing in GFP-expressing NIH3T3 and HeLa cells by determining the fluorescence intensity of the GFP. The GFP fluorescence intensity in NIH3T3 and HeLa cells was decreased by 46% and 58%, respectively [93].

## 4. Delivery of Proteins/Peptides

Peptide and protein cargos have also been frequently delivered into cells by CPPs, where they can exert their biological activity (Table 2) [94]. In comparison with other types of cargos, the conjugation process of peptide cargos can be simpler [34]. Nevertheless, depending on the cell type, the cargo and other variables of peptide applications, the road to success can be challenging. Due to the simplicity of their design and production process, therapeutic peptides and proteins are of specific interest in clinical settings for the treatment of various tumors, but their poor in vivo tumor penetration and sensitivity to degradation restricts their clinical application in cancer therapy [95]. However, in the last decade, several promising results have been achieved in cancer treatment through the utilization of CPPs for the delivery of peptide and protein cargos.

Spinal muscular atrophy (SMA) comprises a group of neuromuscular disorders caused by mutations in survival motor neurons (SMN) resulting in the loss of motor neurons and progressive muscle wasting [96]. Several researches revealed that SMN play vital roles in the assembly of the spliceosome and biogenesis of ribonucleoproteins [97]. The binding of SMN to some Sm proteins (small RNA-binding proteins involved in pre-mRNA splicing) is enhanced by symmetrical dimethylation of arginine residues of an Sm protein. Moreover, a synthetic symmetrical dimethylarginine (sDMA) peptide that contains arginine can inhibit SMN interactions with some Sm proteins such as SmD1, SmD3, SmB and SmB’ [98]. In 2010, Bidwell III et al. designed fusion polypeptides with SynB1 peptide (RGGRLSYSRRRFSTSTGR) as the CPP at the N-terminus, the GRG peptide (GRGRGRGRGRGR) at the C-terminus and a heat-responsive biopolymer carrier named elastin-like polypeptide (ELP), added to enhance cellular uptake and stability via thermal targeting. In particular, the interaction of SMN/SmB was suppressed by the fusion polypeptide. Furthermore, this formulation repressed proliferation and induced apoptosis in HeLa cells [99]. In a similar study conducted two years later, Moktan and Raucher genetically designed a thermally-targeted fusion peptide with ELP, SynB1 and the cationic a-helix forming KLAKLAKKLAKLAK peptide (KLAK) which can induce apoptosis through disturbing the mitochondria. In hormone receptor (HR)-negative and estrogen receptor (ER)-positive human breast cancer cell lines, this formulation exhibited potent cytotoxic activity. Moreover, the thermally-responsive fusion polypeptide enhanced tumor targeting by the application of mild hyperthermia [100]. Additionally, this group also designed a formulation (named Bac-ELP1-p21) consisting of Bac as the CPP at its N-terminus, elastin-like polypeptide (ELP), and the p21^Waf1/Cip1^-derived cell cycle inhibitory peptide. The evaluation of this construct in the ovarian cancer cell line SKOV-3 exhibited considerable suppression of cell cycle-induced apoptosis [101]. Four years later, the polypeptide configuration was reversed to p21-ELP1-Bac, with Bac at the C-terminus and the p21 at the N-terminus of the ELP. Additionally, they assessed this novel construct in pancreatic cancer cell lines and reported enhanced in vitro cytotoxicity and also pronounced tumor growth suppression in an animal model [102]. Key regulators of cell cycle and proliferation such as CyclinD1/CDK4 and cyclinD3/CDK4 complexes can also be considered as valuable targets for the development of various anticancer agents. In this regard, Wang and co-workers conjugated key peptide motifs from these two complexes to protein transduction domain 4 (PTD4). They investigated effects of chimeric peptides in sarcoma/hepatocellular carcinoma xenograft mouse models and reported induced cell cycle arrest, apoptosis, and tumor volume shrinkage [103].

Azurin is a 128 residue periplasmic bacterial blue copper protein found in Pseudomonas aeruginosa [104]. Amino acids 50–77 (p28) of azurin are fundamentally responsible for azurin’s penetration capacity into cancer cells [105]. Mehta and colleagues demonstrated the ability of p28 to penetrate into human umbilical vein endothelial cells (HUVEC), mediating the inhibition of VEGF-induced migration and inducing antiangiogenic effects in multiple xenograft models. The evaluation of p28 in Phase II clinical trials has given rise to promising results including cell cycle arrest and suppression of tumor cell proliferation [106]. In a similar study, Warso et al. demonstrated that p28 binds to the tumor suppressor protein p53 and inhibits its ubiquitination by penetration into the nucleus. As a result, cyclin A1 and CDK2 levels decrease and tumor growth halts in the G2/M stage of the cell cycle. The results of Phase I clinical trials have revealed that p28 is well-tolerated and exhibits remarkable antitumor activity and insignificant toxicity without immunogenicity [107].

The voltage-dependent anion channel 1 (VDAC-1), a, is a mitochondrial porin ion channel located on the outer mitochondrial membrane [108]. VDAC-1 plays a critical role in binding anti-apoptotic proteins such as hexokinase (HK) and the Bcl-2 family and inducing mammalian mitochondrial-dependent apoptosis [109]. In B-cell chronic lymphocytic leukemia (CLL), these anti-apoptotic proteins are frequently overexpressed. Prezma et al. showed that the N-Terminal-Antp and Antp-LP4 (as VDAC1-based peptides) specifically mediate the cytolysis of CLL patients’ peripheral blood mononuclear cells [110]. Additionally, the steroid receptor coactivator-1 (SRC-1), as a transcriptional co-regulatory protein, is also overexpressed in several cancers. In this regard, Tints and coworkers studied the effects of a peptide comprising the LXXLL-motif of the human SRC-1 nuclear receptor box 1 conjugated to the transportan 10 (TP10). This construct rigorously reduced the proliferation and viability of the hormone-unresponsive breast cancer cells MDA-MB-231 [111]. Moreover, one of the valuable targets for the development of anticancer agents is the epidermal growth factor (EGF) receptor which plays a pivotal role in tumor growth and progression [112]. To find a particular inhibitor for this receptor, Abe et al. developed oligopeptides that target auto phosphorylation sites of the EGF receptor. They found two classes of inhibitor peptides: Ac-DYQQD-NH2 and Ac-QNAQYLR-NH2 inhibited phosphorylation of the purified EGF receptors in an ATP-competitive manner, whereas Ac-NYQQN-NH2 and Ac-ENAEYLR-NH2 suppressed it in a non-ATP-competitive fashion. To simplify penetration into human lung carcinoma cells, they linked these peptides to the TAT peptide. The cells treated with the TAT-conjugated peptides exhibited significant inhibition in the EGF-mediated tyrosine phosphorylation of the EGF receptor. Furthermore, TAT-acp-DYQQD-NH2 and TAT-acp-NYQQN-NH2 inhibited the anti-apoptotic effects of EGF whereas Tat-acp-ENAEYLR-NH2 considerably repressed cell proliferation and exhibited cytotoxicity [113]. In the same year, Zhao et al. fused TAT peptide to the gp96 protein to enhance antitumor T cell responses. The heat shock protein gp96 is an adjuvant that can prompt T cell responses against cancer cells. Its uptake and penetration into antigen-presenting cells (APCs) is a vital step in the gp96-mediated immune response. In summary, this recombinant fusion slightly reduced the aggregation level of gp96 and considerably enhanced its penetration into macrophages. rTAT-gp96 remarkably enhanced CTL cytotoxicity and specificity in B16 melanoma C57BL/6 mouse models [114].

The use of potent macromolecular anticancer agents has gained considerable attention because of the poor therapeutic index of small-molecule drugs [115]. One of these agents, Gelonin (30 kDa), is a plant-derived ribosome-inactivating protein (RIP) extracted from the *Gelonium multiflorum* seeds that acts as a toxin to inactivate ribosomes via a single adenine residue (A4324) cleavage in the 28S ribosomal RNA [116]. However, due to the inability of Gelonin to penetrate into cells, it has only demonstrated limited antitumor activities. To overcome the barrier of cell membranes, Shin et al. conjugated Gelonin, via both genetic recombination and chemical conjugation methods, to low molecular weight protamine (LMWP), as the CPP. Further cytotoxicity investigations revealed that the new compound shows considerably enhanced therapeutic effects. In detail, substantial suppression of tumor growth was detected when 2 μg/tumor of this compound was administered into a CT26 s.c. xenograft tumor mouse model [117]. Two years later, the same group chemically synthesized a heparin-functionalized carcinoembryonic antigen (CEA) specific murine monoclonal antibody and evaluated its selective binding capability to CAE-overexpressing cells. Next, in order to devise an effective means of active tumor targeting, a recombinant TAT-gelonin chimera was conjugated to this monoclonal antibody for targeting the CEA-overexpressing cancer cells LS174T. Moreover, the in vivo biodistribution and therapeutic efficacy of this drug delivery system (DDS) were confirmed using LS174T s.c. xenograft tumor-bearing mice [118].

The reduction of the “functional dose” of apoptotic proteins is a common occurrence in a majority of cancers. The low cellular level of the BH3-interacting domain death agonist (BID) protein is a vital factor for the viability of various cancer cells. Thus, one suggested functional strategy for cancer therapy is inducing the overexpression of BID using pcDNA or adenovirus vectors; however, achieving precise control over the cellular level of BID is a difficult task [119]. Through the fusion of the TAT peptide with BID (TAT-BID), Orzechowska et al. generated a construct to induce necrosis factor-related apoptosis-inducing ligand TRAIL-based death in several cancer cells [120]. Plk1 plays critical roles in the regulation of mitotic progression and other cell cycle procedures [121]. Moreover, high mRNA and protein expression levels of Plk1 have been detected in proliferating cells such as tumor cells [122]. Furthermore, several investigations have indicated that targeting the Polo-box domain (PBD) at the C-terminal non-catalytic region of Plk1 can disrupt the catalytic activity of the enzyme. In this regard, the artificial phosphopeptide Pro-Leu-His-Ser-pThr (PLHSpT) has been recognized to be capable of targeting PBD and inducing apoptosis in cancer cells. Additionally, Kim et al. generated a TAT-PLHSpT fusion with high cellular uptake, capable of increasing the suppression of Plk1 kinase activity and reducing cancer cell growth and survival [123].

**Figure 5:**
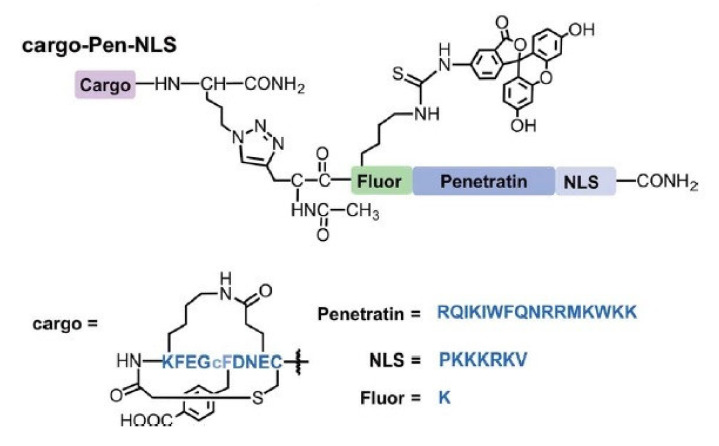
Schematic describing Cargo-Pen-NLS synthetic targets. Amino acid sequences are shown in blue font, while chemical structures, showing non-amino acid functionalities, are represented in black. Adapted with permission from [94], John Wiley & Sons, 2018.

Penetratin (Pen) is one of the first recognized CPPs that has been utilized in multiple investigations. Specifically, the first 16 amino acids of Pen are derived from the third helix of the Antennapedia protein homeodomain [124]. Alves et al. coupled Pen to a pro-apoptotic amphipathic peptide named KLA (acetyl-(KLAKLAK)_2_-NH_2_) through a disulfide bond. In several cancer cell lines and tissues, KLA-Pen displayed potent effects on the mitochondria tubular organization [125]. In 2018, e.g., Kulkarni and colleagues developed a construct composed of the bicyclic peptide inhibitor of growth factor receptor-bound protein 7, known as GRB7, (G7-B7M2), a nuclear localization signal (NLS), and an FITC-labeled Pen (Figure 5). GRB7 plays an important role in the integrin signaling pathway and cell migration. The application of this complex on the breast cancer cell line MDA-MB-231 demonstrated efficient cellular uptake and cytosolic localization without endosomal entrapment. Furthermore, the effective delivery of the G7-B7M2 cargos to the cytosol of a GRB7-overexpressing human breast cancer cell line inhibited the interactions of GRB7 with its upstream binding partners [94].

The advantages of making the anti-apoptosis clone-11 (AAC-11) protein as the target of anticancer therapy were introduced by Koci and co-workers at the beginning of this decade [126]. The anti-apoptotic protein AAC-11 is known to be highly overexpressed in numerous cancer cells and tissues [127]. The functional leucine zipper (LZ) domain of AAC-11 is required for its assembly; hence, with a protein−protein interaction module and inducing inactivating mutations within the LZ domain, its anti-apoptotic and pro-metastatic abilities can be eliminated [128]. Based on this fact, Jagot-Lacoussiere et al. developed a construct called RT53 by the conjugation of the AAC-11 LZ domain, as a competitive inhibitor of AAC-11, to Pen. The study outcomes indicated that RT53 was able to induce apoptosis in cancer cells in a selective manner. Furthermore, RT53 hindered the proliferation of cancer cells in melanoma tumor xenograft mouse models without mediating toxicity [129]. Two years later, the same group revealed that RT53 can mediate immunogenic cell death of cancer cells through the release of danger signals in prophylactic mouse models [130].

One of the main stimulators of cancer cell progression and survival are hypoxia-inducible factors (HIFs) which are transcription factors that respond to hypoxia in the cellular environment [131]. The dysregulation, overexpression or genetic alterations of hypoxia-inducible factor 1-alpha, also known as HIF-1α, have been profoundly associated with cancer biology, which qualifies HIF-1α as a therapeutically valuable target in cancer therapy [132]. Mylonis et al. demonstrated that the ERK-targeted domain (ETD) plays a crucial role in the activation of HIF-1α. The overexpressed variant of ETD comprising the NES-less (IA) mutant forms and the wild-type phospho-mimetic (SE) have been known to trigger the inactivation of HIF-1 in two hepatocarcinoma cell lines [133]. Recently, the same group developed a fusogenic peptide containing these ETD forms and TAT which efficiently entered hepatocellular carcinoma cells, accumulated inside the nucleus, and triggered the mislocalization of the endogenous HIF-1α to the cytoplasm. In addition, a substantial decrease in the activity of HIF-1 and the suppression of the HIF-1 gene expression under hypoxia was reported in this study. Moreover, TAT-ETD peptides that penetrated into the cell nucleus induced apoptotic cell death of the cancer cells that were able to grow under hypoxia [134]. Cell division control protein 42 (Cds42) is a small GTPase of the Rho family that controls cell migration and cell cycle progression [135]. Recently, Tetley et al. developed a lead 16-mer cyclic peptide using in vitro library selection system (CIS display) and generated a cyclic peptide named C1 (PSICHVHHPGWPCWYQ) that binds to Cdc42 with low nanomolar affinity. To facilitate cell penetration, this cyclic peptide was C-terminally conjugated to the nona-arginine motif (R9). This structure displayed encouraging effects in decreasing proliferation and mainly prevented motility and invasion in human lung cancer cells [136].

Cysteine prenylation is a post-translational modification that is exploited by cells to regulate signal transduction and apoptosis. It generally occurs in eukaryotic proteins at a *C*-terminal CaaX box and is facilitated via prenyltransferases [137]. In 2020, Klimpel et al. developed a construct containing a C-terminal CaaX motif based on Ras sequences named as sC18*-KRas4B (GLRKRLRKFRNK-SKTK-CVIM). This construct exhibited intracellular accumulation, interacting with intracellular prenyltransferases which leads to downstream signaling of Ras proteins in pancreatic cancer cells [138]. Several other therapeutic cyclic CPPs have been reported which target the Ras superfamily. Recently, the importance the signaling potential of the dimerization interface of wild type BRAF in oncogenic Ras expressing cells has been revealed and suggested as a site of therapeutic intervention in targeting cancers resistant to ATP competitive medications. Recently, another group designed and synthesized macrocyclic peptides that bind BRAF with high affinity and block paradoxical signaling in malignant melanoma cells occurring through this drug target [139].

The discovery and application of cyclic CPPs is arguably the most important advancement in the CPP field over the past decade, as they have overcome two of the greatest limitations of CPPs—poor proteolytic stability and low cytosolic delivery efficiency [140]. Dougherty et al. conjugated an amphipathic highly active cyclic CPP (CPP9) to stapled peptide Ac-LTFDHYWKQLTS (MDM2 ligand; named as PDI) to overcome low cytosolic delivery efficiencies and make efficient release from the endosome. Stapled peptides have developed as a new class of therapeutic agents to target intracellular protein−protein interactions. This compound acts as an inhibitor against the MDM2-p53 interaction to induce p53-dependent apoptosis in human osteosarcoma SJSA-1 cells [141].

## 5. Delivery of Chemotherapeutics

Chemotherapy is the most common therapeutic option for cancer treatment [142]. Unfortunately, due to the lack of specificity, it often causes various toxicities and side effects in cancer patients [143]. However, besides low tumor specificity and high toxicity, conventional chemotherapy has also other limitations such as rapid circulation clearance and poor water solubility [144,145]. Moreover, the multi-drug resistance of cancer patients can be considered as another drawback of cancer chemotherapy. These drawbacks keep cancer in place as one of the most life-threatening diseases and health-related conditions [146]. To obtain a more efficacious treatment while minimizing side effects, selective delivery systems have been developed by conjugation of chemotherapeutic agents with particular CPPs. The application of CPPs in pre-clinical models began in the year 2000 with using covalently conjugated chemotherapeutic drugs to attain more favorable pharmacokinetic properties that result in efficient cell and tissue penetration [4]. In the past decade, various studies demonstrated that linking chemotherapeutic agents to CPPs can considerably enhance their effective intracellular delivery (Table 3). The conjugation of some chemotherapeutic drugs such as doxorubicin (Dox), docetaxel (DTX), methotrexate (MTX) and paclitaxel (PTX) (Table 4) to CPPs can improve drug biostability, circulation, accumulation and tumor cell membrane penetration capability [16].

**Figure 6:**
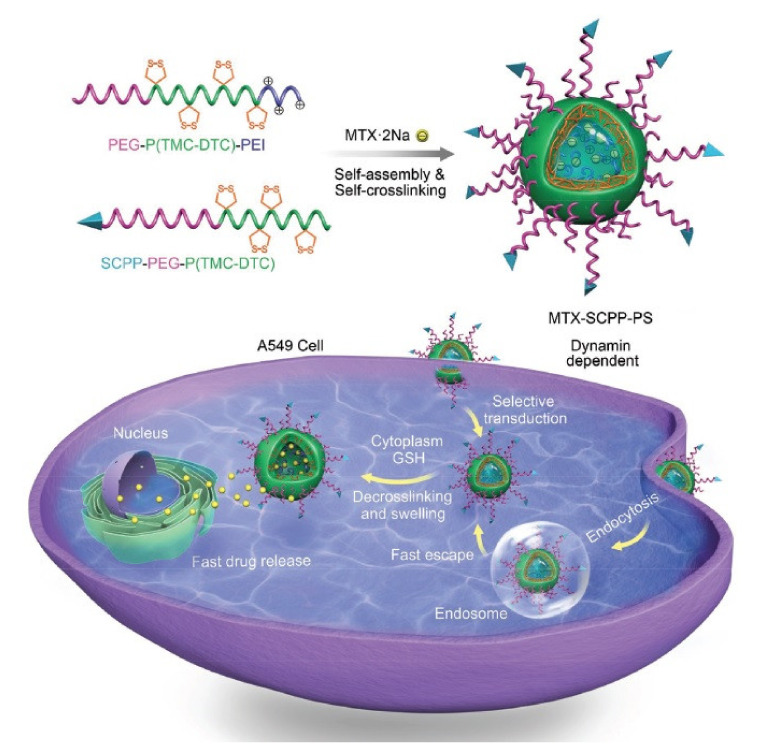
Schematic illustration on fabrication and function of RLWMRWYSPRTRAYGC peptide-decorated polymersomal methotrexate disodium (MTX-SCPP-PS). Adapted with permission from [148], John Wiley & Sons, 2017.

In one study, Szabo et al. conjugated MTX to Pen and investigated the therapeutic impact of this complex on breast cancer cells and reported in vitro cytostatic activity [147]. In another study, Yang et al. introduced a novel peptide as a selective CPP with the sequence RLWMRWYSPRTRAYGC and reported targeted uptake and rapid penetration into lung cancer cells as well as efficient intracellular release of MTX (Figure 6). Furthermore, MTX-loaded (RLWMRWYSPRTRAYGC)-functionalized polymersomes (SCPP-PS) inhibited tumor progression and considerably enhanced the survival rate of A549 lung tumor xenograft-bearing mouse models [148].

**Figure 7:**
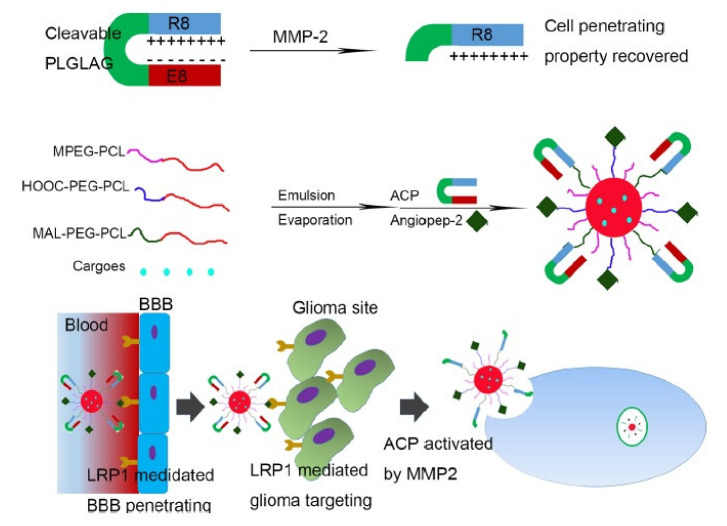
The cell-penetrating property could be activated by MMP-2. AnACNPs could transport through BBB and target glioma because of angiopep-2’s ability to bind LRP1, which is expressed on BBB and glioma cells. Adapted with permission from [151], American Chemical Society, 2014.

To effectively treat glioma without toxicity, multiple delivery strategies have been established. To achieve an improved and targeted delivery, Gao et al. developed a construct containing the AS1411 aptamer and phage-displayed TGN peptide which was loaded with DTX. This formulation facilitated the BBB penetration to reach specific cancer cells and increased the survival rate of glioma-bearing animal models [149]. Two years later, the same group used BBB-penetrating and the activatable cell-penetrating peptide (ACPP) angiopep-2 for the delivery of DTX in glioma-bearing mouse models [150]. For the construction of the ACPP, a matrix metalloproteinase-2 (MMP-2)-sensitive linker was utilized for the conjugation of EEEEEEEE to R8. As mentioned before, Angiopep-2 exhibits high LRP1 binding efficiency and has been used for glioma-targeted delivery by several research groups. This construct exhibited favorable anti-glioma effects both in vitro and in vivo [151] (Figure 7). Recently, in another anti-glioma approach, Kadari et al. used solid lipid nanoparticles covered with angiopep-2 for the delivery of DTX as a targeted drug delivery system [152]. The conjugation of DTX to the polyanionic inhibitory peptide EGGEGGEGG, the MMP-2/9-sensitive cleavable peptide (PVGLIG) and the cell-penetrating domain R9 was associated with enhanced cancer cell-specific uptake of DTX in MMP-2/9-overexpressing tumor tissues. Moreover, this delivery system exhibited pronounced antitumor effects and diminished systemic toxicity in human mouth epidermoid carcinoma mouse models [153].

One of the major limitations of PTX, hampering its therapeutic efficacy as an anti-cancer drug, is its poor solubility profile [154], which can be improved through conjugation to macromolecular carriers. In this regard, Moktan et al. employed the temperature-responsive macromolecular elastin-like polypeptide (ELP) as a carrier for the delivery of PTX. Furthermore, the N-terminus of ELP was modified to harbor SynB1 as the CPP. Through affecting the cells in the G2/M stage, this construction suppressed cell proliferation and induced apoptosis in the MCF-7 cell line [155].

Additionally, the conjugation of tumor-specific pH-responsive peptide H_7_K(R_2_)_2_ (RRK(HHHHHHH)RR) to PLGA-PEG and PTX exhibited remarkable anti-angiogenic and anti-tumor activities in MCF-7 tumor-bearing mouse models in vivo [156]. In another study, TATp-PEG1000-phosphoethanolamine (PE), as a compound that improves cell penetration, was conjugated to MMP2-sensitive micellar nanopreparations combined with PTX. This construct exhibited improved cell internalization, tumor tissue penetration, and antitumor efficacy in monolayer cancer cells and in non-small cell lung cancer xenograft mouse models [157]. PTX has also been applied for glioma treatment by Liu et al. as they developed the multifunctional peptide R8-cyclic RGD. The conjugation of this complex to PTX-loaded liposomes resulted in pronounced growth suppression and apoptosis in C6 cells in addition to inducing higher survival rates in intracranial C6 glioma-bearing mouse models [158]. 

The low molecular weight protamine (LMWP) with sequence of VSRRRRRRGGRRRR is another CPP utilized to enhance the internalization of PTX into the lung cancer cell line A549T. It exhibited elevated cytotoxicity and induced an increase in apoptosis through disrupting the mitosis process [159]. In the same year, Jin et al. developed paclitaxel-loaded nanoparticles linked to bivalent fragment HAb18 F(ab)_2_ and R9, as the CPP, to enhance the therapeutic efficacy of hepatocellular carcinoma chemotherapy. In detail, the drug-loaded nanoparticles exhibited remarkable cytotoxicity against hepatocellular carcinoma both in vitro and in vivo [160].

Among chemotherapeutic drugs, Doxorubicin (Dox) is the most studied agent that has been employed in CPP-based delivery systems (Table 5). Dox, an anthracycline type antitumor agent, has exhibited pronounced antitumor activities in the treatment of malignant lymphomas. However, due to the generation of reactive oxygen species that might lead to the induction of cardiotoxicity, its broader therapeutic application has been hindered [161].

**Figure 8:**
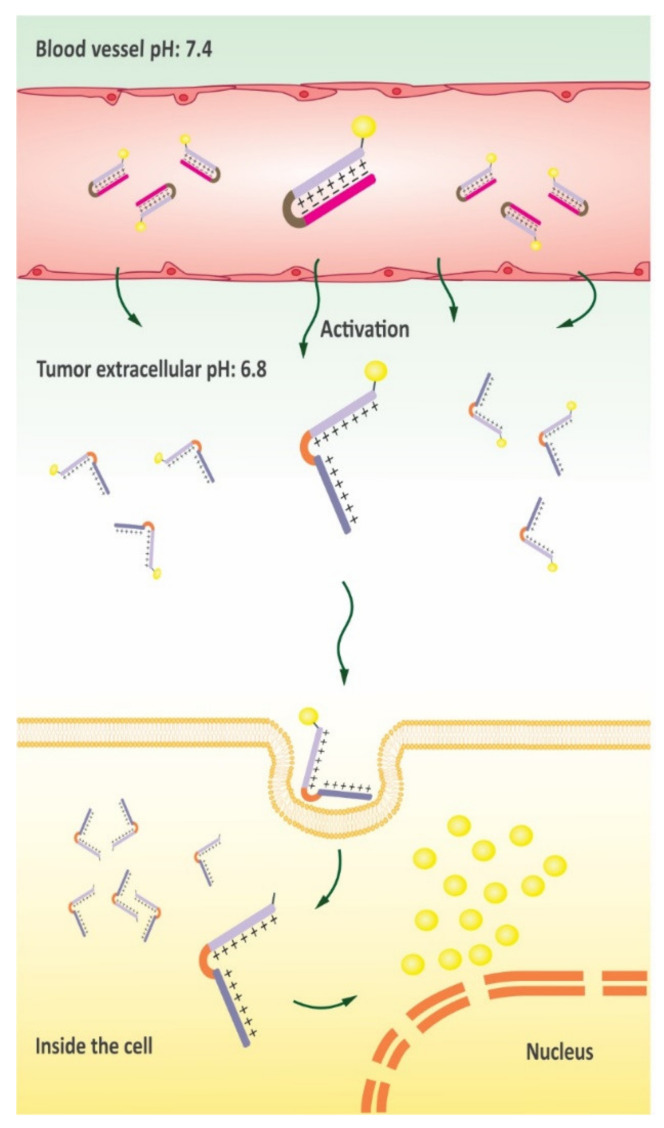
pH would prevent the cell-penetrating function of CPP while acid-triggered hydrolysis of the shielding would activate the cell-penetrating function of CPP for specific cellular uptake and effective antitumor treatment in vivo. The gold bullets represented chemotherapeutics.

To overcome this limitation, multiple efforts have been made to deliver Dox in a less toxic form. For instance, beside minimized side effects, the conjugation of Dox to Pen has induced a cascade of events that result in the release of Cytochrome c, thus triggering cell death by activating intrinsic apoptosis pathway [162]. The self-assembled peptide CADY-1 (GLWWKAWWKAWWKSLWWRKRKRKA) that possesses cell-penetrating activities has also been used for the delivery of Dox. The application of the CADY-1/DOX formulation has resulted in the extension of drug circulation time along with enhanced anti-tumor activity in lymphocytic leukemia xenograft mouse models [163]. In parallel experiments other investigators also evaluated the inhibitory effects of SynB1-ELP-DOX conjugates in breast tumor xenograft mouse models. For example, SynB1-ELP-DOX-induced tumor suppression was 2-fold higher than Dox therapy alone at equivalent doses [164]. In another experiment, Shin et al. designed a conjugate of the activable cell-penetrating peptide (ACPP) DGGDGGDGGDGPLGLAGrrrrrrrrrC and DOX that was sensitive to MMP-2/9. Further experiments showed that the ACPP-DOX cellular uptake was improved after enzymatic-triggered activation, and ACPP-DOX efficiently repressed HT-1080 cell proliferation [165]. As several tumor tissues are characterized with an acidic microenvironment, drug delivery via pH-sensitive biomaterials display significant potential for responsive release of therapeutics triggered via acidic pathological tissues in tumor sites [166] (Figure 8). In a related study, the ACCP CR_8_G_3_PK_6_, with a shielding group of 2,3- dimethylmaleic anhydride (DMA), was conjugated to DOX to build a novel prodrug named DOX-ACPP-DMA for tumor-targeted drug delivery [167].

The progression of multidrug resistance (MDR) in cancer cells is one of the main limitations of cancer chemotherapy [168]. To overcome this caveat, Pan et al. developed a formulation based on decorating mesoporous silica nanoparticles with TAT peptide to generate active nuclear-targeted drug delivery systems. They investigated the efficacy of this construct to efficiently and directly deliver DOX into the nucleoplasm and reported enhanced apoptosis in MCF-7/ADR cancer cells as multidrug resistant models [169]. In terms of tumor-targeted drug delivery and increased drug therapeutic index, various peptide-DOX conjugates have achieved promising outcomes. However, rapid proteolytic degradation has restricted the utilization of peptides in drug delivery [170]. Regarding this matter, Soudy and colleagues used decapeptide 18-4 (WxEYAAQrFL) as a biostable peptide for delivering DOX into breast cancer cells. The results of cell uptake assays indicated that the conjugated complex exhibited a significantly higher specificity for breast cancer cells than for healthy cells [171]. In another experiment, the delivery of DOX by R8-modified PEGylated liposomes (R8-PLD) to non-small cell lung tumor tissues induced higher levels of the apoptotic regulator caspase 3/7 expression, early apoptosis and effective suppression [172]. In a similar study, Sharmay’s group used electrostatic complexation with various polyanionic molecules, such as poly-glutamic acid, hyaluronic acid and heparin sulfate, to enhance the cell specificity of R8. The results demonstrated significantly increased survival rates in mouse models of B16-F10 lung metastasis [173]. The application of CPPs in tumor-targeted nanocarriers has also been extensively explored by various researchers. To achieve a controlled release of encapsulated DOX into the nucleoplasm, Li et al. developed an activatable CPP quantum dot carrier containing TAT, the cathepsin B-responsive linker PGFK, and an inhibitory domain (EEEEEE) [174]. In another study, TAT peptide conjugates to a substrate of endoprotease legumain, alanine–alanine–asparagine (AAN), and liposomes were utilized for targeted delivery of DOX. This formulation enabled liposomal nanoparticles to retain their stability until they were recognized and consequently cleaved by active legumain at tumor sites [175]. As a result of releasing the blocking moiety, liposome internalization was enhanced by the TAT peptide which enabled cell-specific uptake. In a different study, conjugation of TAT-PEG-PE (phosphatidylethanolamine) to commercially available PEGylated liposomal doxorubicin (Lipodox^®^) resulted in enhanced internalization of these nanocarriers into cells along with potentially overcoming MDR effects [176]. In another experiment, Yang et al. used heat-triggered ACPP (CKRRMKWKK)-DOX conjugates to improve selective cancer therapy. Furthermore, thermosensitive liposomes (TSL) comprising NGR peptides as the targeting moiety were also conjugated to this construct. The intravenous administration of CPP-Dox/NGR-TSL resulted in a considerably inhibited tumor progression in fibrosarcoma xenograft mouse models [177]. For a real-time evaluation of drug release dynamics in chemotherapy, in situ monitoring of drug release in cancer cells is highly essential. To this end, DOX-loaded hybrid nanoparticles have been developed in which each nanoparticle contained a cancer cell-targeting antibody, the TAT peptide and gold nanoparticles to enhance the effectiveness of chemotherapeutic drug delivery with selective targeting and improved uptake rate [178]. In a very similar approach, Morshed et al. developed a gold nanoparticle platform surface decorated with TAT peptide to enhance the efficacy of DOX delivery to brain metastatic breast cancer cells. This modified construct induced enhanced cytotoxicity against both a brain and a breast metastatic cancer cell line and caused an increase in the survival rate of a xenograft mouse model of metastatic breast cancer [179]. The cyclic peptide iRGD (CRGDKGPDC) has been shown to be not only a tumor-homing but also a cell-penetrating peptide. This dual function is a result of cell-surface-associated protease activity which causes the neuropilin-1-binding RGDK sequence to become exposed. Using this peptide, Peng and colleagues generated an MMP-2-responsive DOX delivery system for the treatment of prostate cancer. The study outcomes demonstrated that the covalent connection of iRGD via MMP-2 sensitive bonds improves the aggregation and internalization of DOX into tumor cell monolayers and spheroids [180]. In another recent study, cyclic peptides comprising four tryptophan and four arginine residues (C(WR)_4_K) have been used as CPPs conjugated to DOX via disulfide bonds to build a drug delivery system. This delivery system possessed considerably higher cytotoxicity, less unwanted toxicity and pronounced anti-proliferative activity towards mouse myoblast cells in comparison to DOX alone [181]. The potency of poly-l-arginine in improving the cellular uptake of DOX and its cytotoxicity against human prostate cancer cells has been investigated by Movafegh and colleagues [182]. Recently, a conjugate of a lysine-rich CPP (named KRP) and DOX has been introduced as a tumor-targeted drug delivery system. This construct was demonstrated to display enhanced biodistribution and biocompatibility, specific aggregation in tumor sites, a high tendency to stay in tumor tissues, and improved penetration into tumor cells [183].

There are also some other chemotherapeutic agents that have been delivered via CPPs and demonstrated promising in vitro and in vivo results. For instance, Pemetrexed (Pem) is an anti-cancer (antineoplastic and antimetabolite) chemotherapy drug that inhibits enzymes involved in folate pathway, and its application has been associated with encouraging clinical outcomes in several cases of solid tumors [184]. In this regard, Miklán and colleges developed a construction containing Pem, R8 and lung-targeting peptide H-IELLQAR-NH2, which exhibited considerable cytostatic effects on human leukemia cell lines as well as on non-small cell lung carcinoma in vitro [185]. In a similar study, Koshkaryev et al. used R8 for delivery of Bleomycin (BLM) into the cytosol to improve its therapeutic action. In particular, they assembled fusogenic R8-modified DOPE-liposomes conjugated to BLM which exhibited substantially higher DNA damage and apoptosis relative to all control groups as well as pronounced anticancer effects in mammary carcinoma mouse models [186].

Another example is a self-assembling Taxol-CPP (EEGRLYMRYYSPTTRRYG) conjugate. In detail, the anticancer activity of Taxol-CPP was evaluated against human hepatocellular carcinoma cells, which exhibited slightly higher IC_50_ values compared to Taxol treatments alone [187]. As another example, Epirubicin is an anthracycline drug used for chemotherapy. According to one study, TAT-conjugated Epirubicin-loaded poly (lactic-glycolic acid) nanoparticles showed acceptable biocompatibility and substantially enhanced antitumor activity and biodistribution. The clathrin-dependent endocytic and non-toxic CPP KKLFKKILKKL-NH2 or BP16 has been known to be able to penetrate into cancer cells and subsequently accumulate in late endosomes [188]. In this regard, Soler and co-workers evaluated the anticancer efficacy of BP16-chlorambucil (CLB) conjugates and reported increased cytotoxicity with a selective release in lysosomal compartments [189].

One of the major chemotherapy treatments for glioblastoma multiforme is cisplatin-based therapy, however, drug resistance and unwanted side effects have restricted the efficiency of this particular therapy [190]. To develop a more selective cisplatin therapy, Aroui et al. used a highly efficient CPP derived from Tunisian chactid scorpion toxin (L-MCa) for cisplatin delivery. This molecule exhibited improved anti-cancer efficacy compared to plain cisplatin treatment and induced apoptosis in human glioblastoma cells [191]. Furthermore, in an experiment conducted by Izabela et al., TP10-cisplatin conjugates were able to enter tumor cells by a non-endocytic concentration-dependent pathway. It was shown that anti-cancer effects of cisplatin could be considerably improved by TP10 conjugation, as evidenced in human cervical tumoral and osteosarcoma cells [192]. Finally, Gronewold and co-workers demonstrated that actinomycin D conjugation to (sC18)_2_ (derived from the C-terminal domain of the cationic antimicrobial peptide CAP18), as the CPP, increases the cellular uptake and cytotoxic activity of this drug towards human breast adenocarcinoma cells [193]. All these investigations and various other studies indicate that selective delivery of chemotherapeutics utilizing particular CPPs can improve their therapeutic index, making them therapeutically valuable.

## 6. Concluding Remarks and Future Perspectives

**Figure 9:**
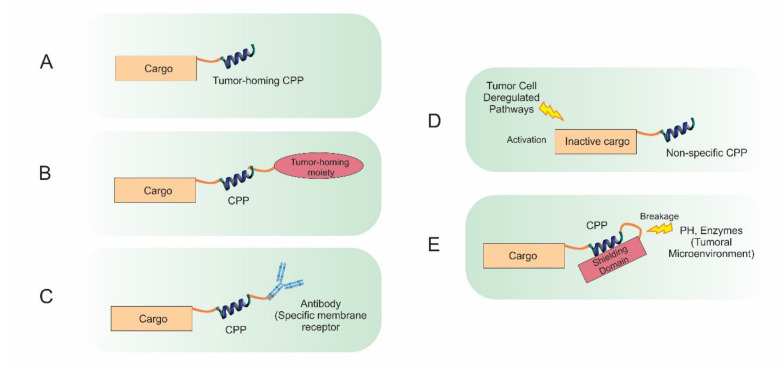
Tumor-specific CPP-conjugate delivery strategies. To additionally improve intracellular uptake of conjugates via CPPs, cargos can be conjugated to either tumor-homing CPPs (**A**), tumor-homing moiety (**B**) or a specific antibody for a membrane receptor (**C**). CPPs can enter any kind of cell, but the cargo is only active inside tumorous cells where molecular pathways are deregulated (**D**). CPP-based drugs can be activated in the proximity of the tumor microenvironment (**E**).

Plenty of evidence provided here shows the key role and value of CPPs in an extensive range of biomedical applications in cancer therapy. In the past few decades, much effort has been devoted to the discovery of novel cancer therapies. CPPs are receiving a great deal of attention due to their ability to deliver large cargos into numerous cancer cells. Despite their significant benefits, several limitations including susceptibility to proteases, uptake into intracellular endosomes and low cell specificity have restricted broader application of CPPs. Although p28 is being investigated in two phase I clinical trials for the treatment of p53 mutated solid tumors, no CPP-based drug has yet obtained US FDA approval for clinical application [194]. In recent years, considerable attempts have been made to overcome CPP limitations by improving their functional efficacy. Poor stability due to proteolysis in physiological fluids is considered as one of the major limitations of current CPPs. In addition, there are negatively-charged sialic acids present on most cellular membranes; thus, most CPPs with net positive charges generally interact with negatively-charged cell surfaces, penetrating into cells regardless of the cell type. Weak penetration as a result of various cellular barriers is another limiting factor for CPP exploitation in biomedical applications. One solution to provide anti-protease shielding is to structurally modify CPP-based delivery systems through conjugating chemical agents. Moreover, significant progress has been made in terms of selectivity (Figure 9). For instance, particular tumor-homing CPPs have been developed by engineering peptides to be sensitive to physicochemical features of tumor sites. Several engineering strategies, such as exploiting ACPP as a shielding strategy, can be adopted to design CPPs which have no function in physiological circumstances but become active once they enter the tumor microenvironments [150]. Furthermore, CPPs can be fused to a targeting moiety or to an antibody molecule to increase their selectivity towards tumor cells based on the recognition of a particular marker expressed on the cell surface. For example, Gaston et al. explored the possibility of genetic fusion of five different CPPs to full-length IgG to serve as a shuttle for antibody targeting into the cytosol of specific cells. Results showed that two out of five tested CPPs considerably improved antibody penetration into the cytosol [195]. Using a natural peptide ligand that binds to an overexpressed receptor on particular tumor cells (such as the RGD peptide), numerous CPPs have been developed with a remarkable selectively and robust affinity for a given target.

Furthermore, the key to successful exploitation of CPPs is to optimize the therapeutic efficacy of CPPs, while developing strategies to minimize their toxicity caused by low specificity following systemic administration. Such strategies have been generally adopted in tumor-affected tissues, which are characterized by particular attributes including hypoxia, low transmembrane potential, proteases overexpression and acidic pH. CPPs have many advantages including affordable production processes, insignificant toxicity, rapid elimination and being feasible candidates for molecular modifications [196]. These factors make CPPs practical tools for clinical applications. However, the low biostability and short plasma half-life of CPPs have limited their application in the delivery of small molecules [197]. In this respect, the conjugation of CPPs to macromolecular carriers such as biopolymers or liposomes along with incorporation of unnatural amino acids serve as promising approaches to enhance their half-life and pharmacokinetic properties. Moreover, the risk of off-target effects and the emergence of resistance mechanisms are other obstacles faced by clinical application of CPP-based chemotherapeutic agent delivery systems. We believe that two main challenges associated with CPP development are their short duration of action and poor cell specificity which require novel technical strategies to overcome and extend the application of CPPs in pharmaceutical and clinical fields.

In conclusion, based on the encouraging outcomes achieved in preclinical investigations of CPPs to date, we are of the opinion that CPPs can be considered as an opportunity for the development of novel anticancer pharmaceutics. The limited experience in peptide-based drug development also makes it difficult to examine pharmacokinetic properties of CPP-based therapeutics in vivo. Nevertheless, the latest developments in designing target-specific peptide sequences, selected through high-throughput screening methods such as phage display, will conquer the future challenges of producing the next generation of CPPs. Moreover, the incorporation of biodegradable bonds proved to be efficient in controlled release and reduced cytotoxicity. Non-natural amino acids comprising azide, alkyne or alkene residues are also incorporated for bio-orthogonal conjugation to improve conformational as well as metabolic stability. The ability to internalize into the cytosol of target cells introduces CPP- based drug delivery systems as potent anticancer therapeutics which could possibly advance into clinical investigations soon and eventually be administered as clinically approved anticancer options.

## Figures and Tables

**Table 1:** Various CPP-based oligonucleotide delivery systems designed for different cancerous cells.

CPP	Cargo	Targeted Tumor	Function	Ref.
**PT_24_**	Cas9	HeLa and the human lung cancer	Delivery of Cas9 in a single incubation step/high efficiency/low toxicity	[43]
**PPABLG**	Cas9	HeLa tumor tissue	Suppressing tumor growth/prolonging animal survival rates	[44]
**TAT**	DNA nanopore	Human Burkitt’s lymphoma	Tumor cell detection with low cytotoxicity	[45]
**TAT**	BCL-2 siRNA	Human breast cancer	Knockdown of the Bcl-2 protein/Inhibition of cancer cell migration	[46]
**R8**	RAC1 siRNA	Human lung carcinoma and ovarian adenocarcinoma	Significantly decreasing the oncogenic RAC1 mRNA levels	[47]
**cRGD**	GFP and Luc mRNA	Human primary glioblastoma	Improving tumor accumulation and potent gene expression	[48]
**RL2**	EGFP siRNA	Human lung adenocarcinoma and epidermoid carcinoma	Substantially decreasing cancer cell viability	[49]
**PepFect 6/** **TP10**	HPRT1 siRNA	Human hepatocellular carcinoma	Significantly reducing the expression of HPRT1 without acute toxicity	[50]
**PepFect 14**	SCOs	HeLa cell line	Significant SCO-mediated splice-correction	[51]
**PepFect 14**	HPRT1 siRNA	Human hepatocellular carcinoma	Induction of the knockdown of endogenous genes	[52]
**PepFect 14/28**	Firefly luciferase siRNA	Glioblastoma	Increasing gene-silencing efficiency	[53]
**gH625**	anti-GFP siRNA	Human triple negative breast cancer	Downregulation of GFP expression	[54]
**CH_2_R_4_H_2_C**	VEGF siRNA	Murine sarcoma	Sufficiently suppressing neovascularization on the tumor surface	[55]
**TP–LyP-1**	ID4-specific siRNA	Human ovarian cancer	Suppressing the growth of established tumors and significantly improving survival	[56]
**TP-iRGD**	TNFα siRNA	Human vestibular schwannomas	Silencing genes and protein secretion	[57]
**R9**	Plk1 siRNA	Human breast cancer	Inhibition of breast tumor growth	[58]
**599 Peptide**	CIP2A siRNA	Oral cancer cells	Significant CIP2A mRNA and protein silencing resulting in the decreasing of oral cancer cell invasiveness	[59]
**TAT-A1**	GAPDH siRNA	Human hepatocellular carcinoma	Decreasing mRNA levels	[60]
**BR2/R9**	VEGF siRNA	Human colon cancer cells/HeLa cells	VEGF silencing/Improving antitumor efficacy without toxicity	[61]

B-cell lymphoma 2 (BCL-2); ras-related C3 botulinum toxin substrate 1 (RAC 1); green fluorescent protein (GFP); enhanced green fluorescent protein (EGFP); hypoxanthine phosphoribosyltransferase 1 (HPRT1); splice-correcting oligonucleotides (SCOs); vascular endothelial growth factor (VEGF); inhibitor of DNA binding 4 (ID4); tumor necrosis factor-α (TNF-α); polo like kinase 1 (PLK1); cancerous inhibitor of protein phosphatase 2A (CIP2A); glyceraldehyde 3-phosphate dehydrogenase (GAPDH).

**Table 2:** Various CPP-based protein and peptide delivery systems designed for various cancerous cells.

CPP	Cargo	Targeted Tumor	Function	Ref.
**SynB1**	ELP1-GRG	Human breast cancer	Disruption of SMN function	[88]
**SynB1**	ELP1-KLAK	Human breast cancer	Mitochondria disruption/Apoptosis induction	[89]
**Bac**	p21-ELP	Pancreatic tumor cells/Human ovarian cancer	Cell cycle inhibition	[90,91]
**PTD4**	cyclin/CDK4 analogs	Murine sarcoma/Hepatocellular carcinoma	Induction of cell cycle arrest and apoptosis	[92]
**p28**	-	Human melanoma cancer/Human colon carcinoma	Inhibition of angiogenesis and tumor growth by inhibiting the phosphorylation of VEGFR-2 and/or p53 ubiquitination	[93,94]
**Antp-LP4/N-Terminal-Antp**	-	B-cell chronic lymphocytic leukemia	Induction of cell death	[95]
**TP10**	LXXLL-motif of the human SRC-1	Breast cancer cells	Induction of dose-dependent cell death	[96]
**LMWP**	Gelonin	Murine adenocarcinoma xenograft tumor	Inhibition of protein translation	[97]
**TAT**	Gelonin/anti-CEA monoclonal antibody	Human colorectal adenocarcinoma	Inhibition of tumor growth	[98]
**TAT**	BID protein	Prostate and non-small human lung cancer	Induction of apoptosis through the TRAIL pathway	[99]
**TAT**	PLHSpT	Human colon adenocarcinoma/Human epidermoid carcinoma	Targeting of Plk1 and the induction of apoptotic cell death	[100]
**TAT**	ETD	Human hepatocellular carcinoma	Inhibition of ERK-dependent activation of HIF-1 and apoptosis triggering	[101]
**Pen**	KLA	Seven human tumor cell lines	Impacting on mitochondria tubular organization/Apoptosis induction	[102]
**Pen**	G7-B7M2	Human breast cancer	Blockade of the interactions of Grb7	[85]
**RT53**	-	Melanoma xenograft tumors/Mouse Fibrosarcoma	Targeting of AAC-11 and the induction of cancer cell death/Induction of immunogenic cell death	[103,104]
**R9**	C1	Human lung cancer	Inhibiting Cdc42, decreasing proliferation, preventing motility and invasion	[105]
sC18*	CaaX motif	Human pancreatic cancer	Affect K-Ras downstream signaling and promote cell death	[106]
**CCP9**	PDI	Human osteosarcoma SJSA-1 cells	Inhibitor against the MDM2-p53 interaction/Induction of p53-dependent apoptosis	[107]
**Peptide 38**	Raf dimers	Malignant melanoma cells	Exhibit anti-proliferative activity and inhibit paradoxical signaling	[108]

Survival motor neuron (SMN); vascular endothelial growth factor receptor 2 (VEGFR2); BH3-interacting domain death agonist (BID); ERK targeted domain (ETD); extracellular-signal regulated kinase (ERK); hypoxia-inducible factor 1 (HIF-1); growth factor receptor bound protein-7 (Grb7); antiapoptotic clone 11 (AAC-11); cell division control protein 42 (Cdc42).

**Table 3:** Various CPP-based chemotherapeutic delivery systems designed for cancerous cells.

Chemotherapeutic	CPP	Targeted Tumor	Function	Ref.
**DTX**	Angiopep-2/R8	Orthotopic glioma	Higher glioma localization	[140]
**DTX**	Angiopep-2	Glioblastoma	Selective targeting with higher accumulation	[141]
**DTX**	R9	Human mouth epidermoid carcinoma	Higher antitumor efficacy and lower systemic toxicity	[142]
**MTX**	Pen/R8	Breast cancer cell	Not mediating in vitro cytotoxic effects	[143]
**MTX**	RLWMRWYSPRTRAYGC	Human lung cancer	Inhibition of tumor progression/Improvement of survival rates	[144]
**Pemetrexed**	R8	Non-small cell lung carcinoma/Human leukemia cells	Higher selective cytostatic effects	[145]
**Bleomycin**	R8	Murine mammary carcinoma	Facilitating BLM interaction with nuclear material	[146]
**Taxol**	EEGRLYMRYYSPTTRRYG	Human hepatocellular carcinoma cells	Efficient cytotoxicity comparable to that of free Taxol	[147]
**Epirubicin**	TAT	Murine hepatic cancer	Improvement of antitumor activity and biodistribution	[148]
**Chlorambucil**	BP16	Breast cancer cell/HeLa cells	Selective release of CLB in lysosomal compartments	[149]
**Cisplatin**	D-MCa	Human glioblastoma cells	Induction of apoptosis by triggering the ROS-ERK/AKT-p53 pathway	[150]
**Cisplatin**	TP10/PTD4	Human osteosarcoma	Nontoxic anticancer activity	[151]
**Actinomycin D**	(sC18)_2_	Breast cancer cells	Decreasing cancer cell viability	[152]

**Table 4:** A list of different CPPs used for paclitaxel delivery to cancerous cells.

CPP	Target cell and tissue	Function	Ref.
**SynB1**	Breast cancer cells	Induction of cell cycle arrest and apoptosis	[153]
**H_7_K(R_2_)_2_**	Breast tumor-bearing nude mice	Inhibition of tumor growth	[154]
**TAT**	Non-small cell lung cancer xenograft mouse models	Tumor growth inhibition/Induction of apoptosis in tumor tissues	[155]
**R8-RGD**	Glioma-bearing mice	Prolonging survival in intracranial C6 glioma-bearing mice	[156]
**TAT and LMWP**	Drug-resistant lung cancer	Influencing mitosis/Inhibition of tumor growth	[157]
**R9**	Hepatocellular carcinoma	Maximization of the therapeutic efficacy for targeting and effective endocytosis	[158]

**Table 5:** A list of different CPPs used for Doxorubicin delivery to various cancerous cells.

CPP	Target Cell and Tissue	Function	Ref.
**Pen**	CHO cells	Initiation of apoptosis through involving c-Jun NH2-terminal kinase	[162]
**CADY-1**	Mouse lymphocytic leukemia	Increasing the blood residence time and/or therapeutic index of the drug	[163]
**SynB1**	Murine breast tumors	Tumor inhibition 2-fold higher than that of free doxorubicin	[164]
**DGGDGGDGGDGPLGLAGrrrrrrrrrC**	Breast cancer/Fibrosarcoma	Antiproliferative effect with less toxicity	[165]
**CR_8_G_3_PK_6_**	Hepatic tumor xenograft mouse models	Significant tumor growth inhibition	[166]
**TAT**	Breast cancer cells	Decreasing cancer cell viability	[167]
**Decapeptide 18-4**	Breast cancer cells	4-fold more antitumor effects towards cancer cells	[168]
**R8**	Non-small cell lung cancer	Induction of higher levels of apoptosis /inhibition of tumor growth/tumor weight reduction	[169]
**P-R8**	Murine model of B16-F10 lung metastasis	Significantly prolonging survival rates in mice	[170]
**TAT**	Lung adenocarcinoma	Increasing Doxorubicin accumulation and inducing higher tumor elimination	[171]
**AAN-TAT**	Breast cancer cells	Limited toxicity/Increasing the tumoricidal effects of doxorubicin	[172]
**TAT**	Drug-resistant ovarian cancer models	Enhancing cytotoxicity in drug-resistant cells	[173]
**NGR peptide**	Fibrosarcoma xenograft mouse models	Inhibition of tumor growth	[174]
**TAT**	Brain metastatic breast cancer	Improving survival rate in xenograft mouse models	[175]
**iRGD**	Prostate cancer cells	Enhancing the accumulation and penetration of doxorubicin	[176]
**C(WR)_4_K**	Human ovarian cancer/Fibrosarcoma	Localization in the nucleus/reduction of toxicity	[177]
**Poly-L-arginine**	Human prostate cancer	Facilitating doxorubicin uptake and increasing its intracellular concentration	[178]
**KRP**	Human osteosarcoma cell	Pronounced biodistribution/Selective accumulation in tumor tissues	[179]
